# Regulation of GC box activity by 8-oxoguanine

**DOI:** 10.1016/j.redox.2021.101997

**Published:** 2021-04-30

**Authors:** Nadine Müller, Andriy Khobta

**Affiliations:** a Unit “Responses to DNA Lesions”, Institute of Toxicology, University Medical Center of the Johannes Gutenberg University Mainz, Mainz, 55131, Germany; b Institute of Nutritional Science, University of Jena, Jena, 07743, Germany

**Keywords:** 8-Oxoguanine, 8-Oxoguanine DNA Glycosylase (OGG1), AP lesion, GC box, Base excision repair (BER)

## Abstract

The oxidation-induced DNA modification 8-oxo-7,8-dihydro-2′-deoxyguanosine (8-oxodG) was recently implicated in the activation and repression of gene transcription. We aimed at a systematic characterisation of the impacts of 8-oxodG on the activity of a GC box placed upstream from the RNA polymerase II core promoter. With the help of reporters carrying single synthetic 8-oxodG residues at four conserved G:C base pairs (underlined) within the 5′-TGGGCGGAGC-3′ GC box sequence, we identified two modes of interference of 8-oxodG with the promoter activity. Firstly, 8-oxodG in the purine-rich (but not in the pyrimidine-rich) strand caused direct impairment of transcriptional activation. In addition, and independently of the first mechanism, 8-oxodG initiated a decline of the gene expression, which was mediated by the specific DNA glycosylase OGG1. For the different 8-oxodG positions, the magnitude of this effect reflected the excision preferences of OGG1. Thus, 8-oxodG seeded in the pyrimidine-rich strand was excised with the highest efficiency and caused the most pronounced decrease of the promoter activity. Conversely, 8-oxodG in the symmetric position within the same CpG dinucleotide, was poorly excised and induced no decline of the gene expression. Of note, abasic lesions caused gene silencing in both positions. By contrast, an uncleavable apurinic lesion in the pyrimidine-rich strand enhanced the GC box activity, suggesting that the AP endonuclease step provides a switch between the active versus repressed promoter states during base excision repair.

## Highlights

• 8-oxodG in GC box affects the promoter activity in a position-dependent manner.
• OGG1 and APE1 govern the outcomes of 8-oxodG for the GC box activity.
• The efficiency of the OGG1 step defines the magnitude of transcriptional response.
• APE1 acts as a switch between the activation and repression.

## Graphical abstract

**Figure:**
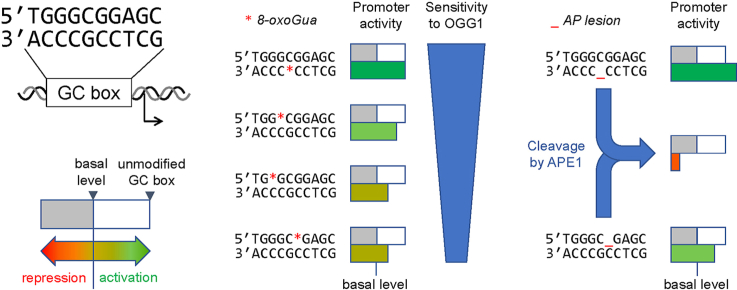

## 1 Introduction

8-oxo-7,8-dihydroguanine (8-oxoGua, commonly referred to as 8-oxoguanine) is a major product of guanine oxidation in genomic DNA caused by reactive oxygen species (ROS) of both endogenous and exogenous origin. The number of 8-oxodG lesions formed per cell of human body per day exceeds 1000 [1]. In cells with normal DNA repair function this translates into a steady-state level of 500–2000 oxidised guanine lesions (dominated by 8-oxodG) in the genome, and this load can be multiplied under oxidative stress or when repair is compromised [2,3]. If unrepaired, 8-oxodG causes characteristic C > A transversion mutations, which arise via a non-canonical (Hoogsteen type) base pairing with adenine during the replication of DNA [4,5] and define one of the common mutation signatures found in human cancers [6]. An elaborate complex of defence mechanisms known as “GO system”, including the 8-oxodGTP hydrolase MTH1 and two DNA N-glycosylases (OGG1 and MUTYH) with different base excision repair activities, has evolved to protect the genome from the mutagenic effects of 8-oxodG [7]. In human cells, base excision repair (BER) initiated by the specific 8-oxoguanine DNA glycosylase (OGG1) constitutes the first line in this multi-layered defence system. OGG1 recognizes 8-oxodG paired with C and excises it to generate either a 3′-α, β-unsaturated aldehyde or an apurinic (AP) sugar residue. In the subsequent step, apurinic/apyrimidinic site nuclease (APE1) cleaves the phosphodiester linkage 5′ to the primary excision product to enable the downstream BER reactions, which typically involve DNA polymerase beta and a DNA ligase [8,9].

The discovery of a highly conserved pattern of distribution of 8-oxodG in the genome suggested a non-stochastic character of the damage generation or/and variable repair efficiencies along human chromosomes [10]. Recent advances in the whole-genome sequencing techniques allowed a higher resolution mapping of 8-oxodG over the genomes of various organisms which revealed highly heterogeneous 8-oxodG distribution patterns [[11], [12], [13], [14], [15], [16]]. Intriguingly, 8-oxodG clustering in the specific chromosomal regions showed pronounced correlations with both chromatin organisation [11,12] and functions of the identified genome elements in DNA replication and transcription [[12], [13], [14], [15], [16]]. The observations of sharp 8-oxodG distribution patterns corroborate the notion that, at least, some portion of genomic 8-oxodG might be generated in a regulated fashion and that the abundance of this DNA modification at particular genomic loci is controlled by differential DNA repair [17]. The maintenance of 8-oxodG at specific loci, together with accumulating evidence of its dynamics in response to stimuli, strongly suggest a putative regulatory role of 8-oxodG in the genome function [[17], [18], [19], [20]]. In particular, activation of the gene transcription by a number of transcription factors has been associated with both localised oxidative damage to DNA [[21], [22], [23], [24]] and specific OGG1 recruitment to the target sites in gene promoters [[25], [26], [27], [28], [29], [30]].

The presence of 8-oxodG in DNA may affect transcription via multiple mechanisms [31,32]. The effects induced directly by 8-oxodG include interference with binding of transcription activators/repressors to the cognate DNA sequences [[33], [34], [35]] and modulation of DNA folding into non-B structures [36,37]. Also, the downstream molecular events and their outcomes for the gene transcription can vary strongly among different promoters. Thus, OGG1 can either impede [38] or promote [25,26,29,37] the interaction of transcription factors with their target sequences, depending on the promoter context. Transcription of some genes can be induced by a non-productive binding of OGG1 [25,26,29,37]; others may require 8-oxoGua excision and generation of an AP site for the activation [[39], [40], [41], [42]]. In turn, further cleavage of the AP residue by APE1 can lead either to transcriptional activation [27,43] or repression [39,41,44], adding another complexity level to the mechanisms of transcriptional regulation by 8-oxodG. In GC-rich regulatory elements, some of the observed responses were assigned to the regulation of DNA folding into non-canonical structures by 8-oxodG, AP lesions and proteins bound to these modifications [36,37,39,45].

To rationally interpret the complex effects of 8-oxodG on the function of promoters containing multiple transcription factor binding sites, it is important first to investigate its impacts on the activity of individual regulatory motifs, commonly present in the GC-rich promoter sequences. We chose GC box to dissect the functional consequences of guanine oxidation in a standalone transcription regulatory element, because this motif is recurrently present in GC-rich promoters whose structure and activity are regulated by 8-oxoGua [[39], [40], [41], [42]]. Differently from the previous studies, we investigated the strand-specific effects of 8-oxodG in the promoter sequence without changing the direction of the key upstream regulatory element with respect to the transcription start. By systematically replacing guanine residues at four highly conserved G:C/C:G base pairs within the 5′-TGGGCGGAGC-3′ GC-box consensus sequence with 8-oxodG, we discovered fundamentally different modes of action of 8-oxoGua in the purine- and pyrimidine-rich strands of the GC box and characterised quantitative differences between the three positions within the G-rich DNA strand. Moreover, by interfering with the OGG1 and APE1 steps, we separated the direct impact of 8-oxoGua on the promoter activity from the BER-mediated responses.

## 2 Material and methods

### 2.1 Cell lines

The HeLa-derived cell line, in which the expression of OGG1 DNA glycosylase was stably knocked down by expression of the specific shRNA (OGG1-sh), was described previously along with the corresponding congenic OGG1-proficient HeLa-pEpS cell line (Ctrl-sh) stably transfected with the empty pENTR/pSuper + expression vector (Addgene, Cambridge, MA) [46]. The OGG1-sh cell line retains 30% of the OGG1 protein level (and one third of the specific BER activity) of the “Ctrl-sh” or the parental HeLa cells. The HeLa-derived cell line with stable overexpression of a functional OGG1-GFP fusion protein [47] was obtained from Pablo Radicella (CEA, Fontenay-aux-Roses).

### 2.2 Reporter vectors for assessment of GC box-driven gene transcription

A pair of reporter vectors expressing the enhanced green fluorescent protein (EGFP) gene under the control of a GC box (as the only *cis*-regulatory element present upstream from the RNA polymerase II transcription initiation site) was generated by subcloning of a consensus SP1 transcription factor binding sequence 5′-TGGGCGGAGC into the previously described pCRE-UNO-ZA-W and pCRE-UNO-ZA-C vectors to replace the cAMP response element (CRE) sequences between the BsrDI sites in the vectors of origin [44]. Thereby, proximal promoter regions of the obtained pGCbox-W and pGCbox-C vectors contain a standalone GC box flanked by two tandem sites for the Nb.BsrDI nicking endonuclease in the orientations suited for selective nicking (and replacement) of either the purine-rich or the pyrimidine-rich DNA strand (Supplementary Fig. S1). Note that the GC box motif has the same orientation in both reporter vectors.

### 2.3 Generation of reporter constructs containing 8-oxodG or AP lesions at defined positions in the GC box

**Fig. 1:**
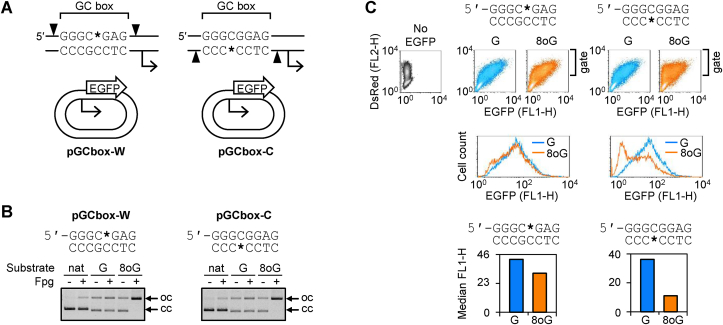
Targeted incorporation of 8-oxodG into opposite DNA strands at the central CpG dinucleotide of a GC box and the outcomes for the expression of the EGFP reporter gene. (A) Scheme of the pair of reporter vectors expressing EGFP under the control of a GC box as the only upstream regulatory element. Arrowheads indicate tandem Nb.BsrDI nicking sites, present at an 18-nucleotide interval in either the purine-rich (pGCbox-W) or the pyrimidine-rich (pGCbox-C) DNA strand. The fragments excised by Nb.BsrDI were substituted with synthetic oligonucleotides containing either dG or 8-oxodG at the positions marked with the wildcard (*). Transcription start sites and the direction of transcription are indicated with broken arrows. Note the same direction of the GC box sequence in both vectors. Full sequences of the promoter regions and the gene expression levels are reported in Supplementary Fig. 1. (B) Verification of 8-oxodG incorporation into the reporter constructs by Fpg-mediated conversion of covalently closed vector DNA (cc) into the open circular form (oc). The first two lanes of the gels are native plasmids (nat) prior to incorporation of synthetic strands; next lanes are constructs containing dG/8-oxodG at the indicated positions. (C) Flow cytometry analyses of expression of the reporter constructs containing dG/8-oxodG in HeLa cells 48 h post-transfection (representative result of five independent experiments). Cells were co-transfected with the indicated constructs and the expression vector encoding DsRed-Monomer as a transfection marker. The FL1-H/FL2-H scatter plots (above) were gated selectively for transfected cells to derive the EGFP fluorescence distribution plots (underneath, note the left shifting due to the presence of 8-oxodG). Bar charts (below) report median EGFP fluorescence as a quantitative readout of the gene expression [51].

To incorporate synthetic 8-oxodG/AP lesions into a chosen DNA strand of the GC box sequence, the Nb.BsrDI nicking endonuclease (NEB GmbH, Frankfurt am Main, Germany) was used to generate nicks on both sides of the GC box. The vectors contain no other Nb.BsrDI site except these two. By choosing the appropriate vector (pGCbox-W or pGCbox-C), either the purine-rich or the pyrimidine-rich strand of the GC box was selectively cut by Nb.BsrDI (Fig. 1A and Supplementary Fig. S1). The excised DNA fragments were fully depleted to generate 18-nucleotide gaps in one or another DNA strand by heat denaturation and re-annealing in the presence of a 900-fold excess of the complementary synthetic 18-mers. Circular gapped DNA was cleaned up using Amicon Ultra Ultracel 30 centrifugal concentrators (Millipore, Schwalbach am Taunus, Germany). Finally, the matched synthetic 18-mer oligonucleotides containing dG, 8-oxodG or the indicated types of synthetic AP lesions at the specified positions were seamlessly ligated to seal the gaps (Supplementary Figs. S2 and S3). All reactions were performed as described previously [48], but with different synthetic DNA strands. Complementary oligonucleotides used for generation of single-stranded gaps were: 5′-CGCTCCGCCCATGCAATG (depletion of the purine-rich strand in pGCbox-W) and 5′-CGTGGGCGGAGCGCAATG (depletion of the pyrimidine-rich strand in pGCbox-C). The oligonucleotides used for the incorporation of 8-oxodG or dG at the positions underlined in sequences were: 5′-CATTGCATGGGCGGAGCG, 5′-CATTGCATGGGCGGAGCG, 5′-CATTGCATGGGCGGAGCG (pGCbox-W) and 5′-CATTGCGCTCCGCCCACG (pGCbox-C). The synthetic AP lesions used were tetrahydrofuran with either the phosphodiester (F) or the APE1-resistant phosphorothioate 5′-linkage (SF). The oligonucleotides used for incorporation of the apurinic lesions were: 5′-CATTGCATGGGC[F/SF]GAGCG (pGCbox-W) and 5′-CATTGCGCTCC[F/SF]CCCACG (pGCbox-C). The synthetic strands containing 8-oxodG, F and SF lesions (all HPLC-purified and validated by electrospray ionisation mass spectrometry) were from BioSpring GmbH (Frankfurt am Main, Germany). All the other oligonucleotides were from Eurofins MWG Operon (Ebersberg, Germany).

The presence of 8-oxodG in the covalently closed vector DNA was verified by excision analysis using formamidopyrimidine DNA glycosylase (Fpg) of *E. coli* (NEB GmbH, Frankfurt am Main, Germany). This bifunctional enzyme has DNA N-glycosylase activities towards damaged purines, including 8-oxoG, and the AP lyase activity, which cleaves the arising AP site to generate a single-nucleotide gap. DNA (100 ng/15 μL) was combined with 0.5 units Fpg in the BEH buffer composed of 10 mM HEPES (pH 7.5), 200 mM NaCl, 1 mM ethylenediaminetetraacetic acid, supplemented with 0.1 mg/mL nuclease-free bovine serum albumin (BSA) (NEB). Reactions were incubated 1 h at 37°C followed by heat-inactivation 20 min at 65°C. The presence of the AP lesion and the effect of the adjacent phosphorothioate linkage on the strand cleavage were verified by incubation with 4 units endonuclease IV (NEB) under analogous conditions, but the enzyme was inactivated at 85°C. To determine percentages of covalently closed DNA in the vector preparations and to monitor the incision rates, the reaction products were analysed by agarose gel electrophoresis in the presence of 0.5 mg/L ethidium bromide, as described previously [49].

### 2.4 Quantitative analyses of EGFP expression in transfected cells

Cells exponentially growing in 6-well plates were transfected with 400 ng of the GC box reporter constructs containing the specified modifications in combination with 400 ng of the tracer pDsRed-Monomer-N1 vector (Clontech, Saint-Germain-en-Laye, France) using Effectene (QIAGEN, Hilden, Germany), as described previously [50]. When several time points had to be analysed, transfected cells were split 6 h after transfection. One part was fixed immediately, whereas the rest was re-seeded into separate wells for harvesting at the indicated time intervals. Harvested cells were formaldehyde-fixed to obtain single-cell suspensions and analysed using a FACSCalibur™ flow cytometer and the CellQuest™ Pro software (Beckton Dickinson GmbH, Heidelberg, Germany). EGFP expression was quantified as median FL1-H fluorescence after selective gating of viable transfected cells marked by the concomitant DsRed expression (FL2-H), as described previously [51]. For assessment of inter-experimental reproducibility, relative expression levels were calculated in individual experiments for each type of modification (8-oxodG, F, SF) by normalisation to median EGFP fluorescence of cells transfected in parallel with the reference construct containing dG at the same position.

### 2.5 Quantification of the position-dependent excision efficiencies of 8-oxodG and AP lesions

For the analysis of the OGG1 capacity to excise 8-oxoGua from the GC box sequence, constructs containing single synthetic 8-oxodG residues at the specified positions (150 ng covalently closed circular DNA per 15 μL reaction) were incubated with a concentration range of recombinant human OGG1 (NEB, product discontinued) in NEBuffer4 (NEB) for 1 h at 37°C, either in the absence or in the presence of 1.5 units APE1 (NEB). The reactions were stopped by addition of 6 μL purple loading dye (NEB) containing 0.08% SDS followed by heat-inactivation for 20 min at 65°C.

The APE1 incision analyses at the synthetic abasic lesions (F or SF) were performed in the magnesium-free BEH buffer supplemented with 0.1 mg/mL BSA or in NEBuffer4 (NEB) containing 10 mM magnesium. Constructs containing F, SF or dG at the specified positions (150 ng per 15 μL reaction) were incubated with the specified amounts of APE1 (NEB) for 1 h at 37°C. The reactions were stopped by addition of 4 μL SDS-containing purple loading dye (NEB) and heat-inactivation 20 min at 65°C. The proportions of covalently closed (cc) and open circular (oc) plasmid DNA were determined by a GelDoc™ EZ imager and the Image Lab™ software (Bio-Rad Laboratories, GmbH, Munich, Germany). The detected band intensities were corrected for the different ethidium bromide fluorescence yields of the topologically different forms of circular DNA (cc:oc relate as 1:2.4), as described previously [46].

### 2.6 Analyses of the incision of 8-oxodG and AP lesions by cell lysates

Exponentially growing cells (HeLa or the derived OGG1-GFP overexpressing cells, as specified) were harvested on ice in phosphate buffered saline (PBS) supplemented with 0.5 mM phenylmethanesulfonyl fluoride (Carl Roth GmbH, Karlsruhe, Germany) and centrifuged (4000×*g*, 5 min, 4°C). Cell pellets were resuspended in 0.5 mL of lysis buffer (20 mM Tris-HCl pH 8.0, 1 mM EDTA, 250 mM NaCl) supplemented with cOmplete™ protease inhibitor cocktail (Roche Diagnostics GmbH, Mannheim, Germany). The samples were sonicated on ice-water slurry using an UP200Ht ultrasonic homogeniser (Hielscher Ultrasonics GmbH, Tetlow, Germany) equipped with a microtip. Two 40 s pulses were applied with a 60 s interval at a 10% power setting (40% cycle, 20% amplitude). The insoluble material was removed by centrifugation (21000×*g*, 20 min, 4°C), supernatants were transferred, and the centrifugation step was repeated. Concentrations of the whole cell lysates were equilibrated based on the A_280_ absorbance determined using a Nanodrop 2000™ spectrophotometer (Thermo Fischer Scientific Inc., St. Leon-Rot, Germany) and aliquots were stored at −80°C for single usage. For the incision reactions, 10 ng/μL vector DNA containing the specified modifications were incubated with the specified amounts of cell extract for 1 h at 37°C in BEH-BSA buffer. The reactions were stopped by addition of 4 μL SDS-containing loading dye (NEB), heated for 3 min at 50°C and analysed by electrophoresis in ethidium bromide-containing agarose gels. The incision rates were determined as increased fraction of DNA in the open circular form.

## 3 Results

### 3.1 8-oxodG in the central CpG dinucleotide has a negative impact on the GC box-driven transcriptional activity

To dissect the effects of 8-oxodG in a GC-rich regulatory element in a simplified, well defined system, our strategy was to generate a reporter gene under the control of an upstream regulatory element consisting of a standalone GC box, suitable for targeted incorporation of synthetic 8-oxodG. Previously, we had efficiently used a similar strategy to investigate the effects of 5-methylcytosine oxidation products on the gene expression driven by the transcription factor CREB in an artificial promoter regulated by the cognate CRE motif as the only upstream regulatory element [44]. In these CRE-UNO constructs, the transcription factor binding site was positioned between tandem sequences targeted by the Nb.BsrDI nicking endonuclease, which allowed a seamless integration of modifications of choice into vector DNA by nicking at the two positions followed by exchange of the excised fragment for a matched synthetic oligonucleotide. To create reporters suitable for analysis of the outcome of 8-oxodG in a minimal promoter consisting of a single GC box, we substituted the CRE sequence in the available reporter vectors with a GC box consensus sequence (Supplementary Fig. S1). As both directions of the asymmetric GC box sequence are common in human promoters [52], we chose the direction found in the upstream regulatory region of the DHFR gene, where its activating function was comprehensively characterised [53]. We chose 5′-TGGGCGGAGC as a fully functional GC box sequence [54,55], which is less prone to non-canonical folding or potential misannealing than GC-only sequences with equivalent activities.

The resulting two vectors, designated pGCbox-W and pGCbox-C, contain BsrDI sites in different orientations, whereas the GC box direction with respect to the transcription start site is the same (Fig. 1A and Supplementary Fig. S1). An advantage of such a design is the possibility of inserting a synthetic 8-oxodG either into the purine-rich or the pyrimidine-rich DNA strand. This is different from previous studies of various GC-rich promoter elements, where the effects of 8-oxodG were only addressed within the G-runs of potential G-quadruplex (G4) forming sequences [18,36,39]. Next, we verified the GC box function in the pGCbox-W and pGCbox-C reporter vectors by transfection into HeLa cells. Regardless of the orientation of the BsrDI sites, GC box enhanced the expression of the downstream EGFP gene by a factor of >2 with respect to the background expression level, thus confirming its activatory function in both reporter vectors (Supplementary Fig. 1).

To incorporate synthetic 8-oxodG (or dG as a control) into the central CpG dinucleotide of the GC box in either DNA strand, we generated tandem nicks in both pGCbox-W and pGCbox-C vectors and exchanged the excised DNA fragments with the synthetic oligonucleotides of the matched sequence, as explained in Methods section (see also Supplementary Fig. S2). The incorporation efficiencies of 8-oxodG were practically 100% in both DNA strands, as confirmed by incision of the resulting constructs with the specific Fpg DNA glycosylase (Fig. 1B). The full conversion of the vector DNA into the nicked form also indicated that the structural conformation of the GC box motif in the presence of 8-oxodG allows efficient damage recognition by Fpg. The same holds true also for human OGG1, as discussed in subsequent sections.

We used the obtained reporter constructs to investigate whether 8-oxodG at the indicated positions has an effect on the promoter activity. Expression analyses of 8-oxodG- versus dG-containing constructs in transfected HeLa cells showed that 8-oxodG negatively affects the gene expression (Fig. 1C). The magnitude of the observed effect was higher when 8-oxodG was placed in the pyrimidine-rich strand; however, 8-oxodG in the purine-rich strand also caused a clear impairment of the promoter activity. Thus, in the minimal promoter consisting of a single GC box, the presence of 8-oxodG residues in either DNA strand invariantly led to a decreased promoter activity, in contrast to the alternating activation or repression reported by others in the context of more complex promoter structures [36,37,39].

### 3.2 Transcriptional inhibition by 8-oxodG in the pyrimidine-rich strand but not in the purine-rich strand is OGG1-dependent

**Fig. 2:**
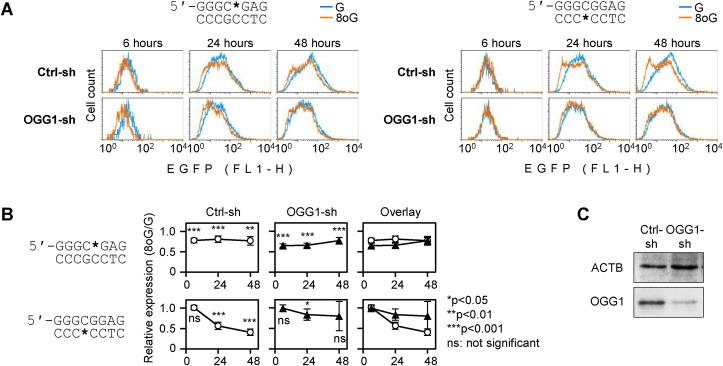
Impact of BER on the effects of 8-oxodG in the central CpG dinucleotide of the minimal GC box promoter on the gene expression. EGFP expression time course in OGG1 proficient (Ctrl-sh) and OGG1 knock down (OGG1-sh) HeLa-derived cell lines. (A) Representative fluorescent distribution plots of cells transfected with constructs containing dG/8-oxodG at the indicated positions (*). (B) Quantification of the expression of constructs containing 8-oxodG, relative to the respective dG constructs (mean ± SD, n = 7). P-values: Student's heteroscedastic, two tailed *t*-test. (C) Assessment of the OGG1 protein levels in exponentially growing Ctrl-sh and OGG1-sh cells by Western blotting with EPR4664(2) rabbit monoclonal antibody, re-probed with C4 antibody (sc-47778) to ACTB as a loading control.

Reports of our and other groups previously suggested that BER of various base modifications and of the derived apurinic/apyrimidinic (AP) lesions, or perhaps non-productive recruitment of the BER components to the lesions, may act in different promoters as an “on” or “off” switch for transcriptional activation [37,39,44]. We, therefore, analysed the expression of 8-oxodG-containing GC box reporter constructs in an isogenic pair of cell lines with different OGG1 expression statuses. A stable OGG1 knockdown HeLa cell line and the cognate cell line stably expressing a non-targeting short RNA were generated and phenotypically validated in our laboratory previously [46]. We monitored the EGFP expression in both cell lines over a 48-h time course and found that outcomes of 8-oxodG situated in opposite DNA strands differed strikingly, depending on the presence of OGG1 in cells. When positioned in the purine-rich strand of the GC box sequence, 8-oxodG caused a decrease of the EGFP expression to the levels between 64.5 ± 4.3% and 80.6 ± 7.8% of the dG control. At all time points taken between 6 and 48 h post transfection, the expression differed significantly from the control dG construct (p < 0.01) but was similar in the OGG1-deficient and -proficient cell lines (Fig. 2). In contrast, the responses to 8-oxodG in the pyrimidine-rich strand of the GC box were notably different, depending on the OGG1 status. In the OGG1-knockdown cell line, the EGFP expression was barely influenced by 8-oxodG at any time point, whereas the OGG1-proficient cell line clearly displayed a gradual time-dependent decline of the EGFP expression. The EGFP signal from the construct containing 8-oxodG did not yet differ from the control dG construct at 6 h after transfection. However, it underwent a very significant (p < 0.001) decrease at the subsequent 24-h and 48-h points, thereby indicating that 8-oxodG in the pyrimidine-rich strand of the GC box induced repression of the gene transcription in an OGG1-dependent manner. Based on the observed results, we conclude that 8-oxodG residues situated in different stands of the central CpG dinucleotide of the GC box sequence affect the gene expression by two distinct mechanisms. In the purine-rich strand, 8-oxodG directly inhibits the promoter activity, albeit to a relatively small degree. In contrast, in the pyrimidine-rich strand, 8-oxodG per se seems to be neutral for the promoter activity. However, in the presence of OGG1, it has a potential to induce transcriptional repression.

### 3.3 DNA strand specificity of transcriptional repression by 8-oxodG correlates with the incision preference of OGG1

**Fig. 3:**
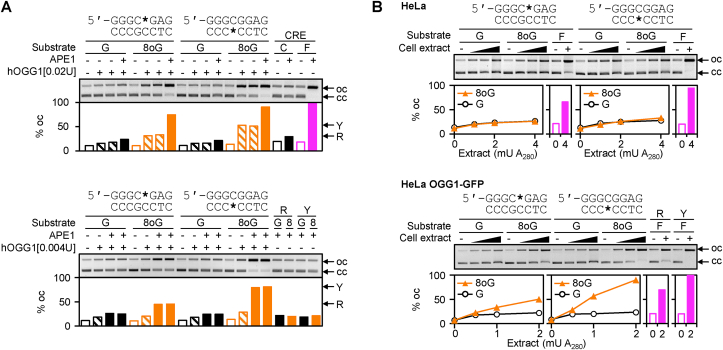
Efficiencies of 8-oxodG processing in opposite strands of the central CpG dinucleotide of the GC box by human BER. (A) Incision of constructs containing dG or 8-oxodG at the indicated positions (*) by pure OGG1 in the presence (1 unit/100 ng DNA) or in the absence of APE1. Covalently closed circular DNA substrates (cc) were incubated with either 0.02 units (upper gel and quantification underneath) or 0.004 units (lower gel) of human OGG1 (hOGG1). Incision efficiencies were measured as percentages of conversion into the open circular form (oc). Note that both in the absence of APE1 (hatched bars, independent duplicates in the upper gel) and in its presence (filled bars, independent duplicates in the lower gel), the pyrimidine-rich DNA strand (Y) is incised more efficiently than the purine-rich strand (R). The last four lanes in the upper gel show a proof of the APE1 activity: complete incision of an unrelated sequence (CRE) containing a synthetic tetrahydrofuran AP lesion (F) and the absence of nicking of the matched dC construct (C) as the negative control [44]. The last four lanes in the lower gel show that APE1 by itself does not nick the GC box substrates containing dG (G) or 8-oxodG (8) in either strand (“R” or “Y”) under the chosen conditions. Representative results of one of three reproducible experiments for both conditions. (B) Incision efficiencies of 8-oxodG in the pyrimidine- and in the purine-rich DNA strands (“R” and “Y”) by HeLa protein extract (upper panel) or the extract of the derived cell line overexpressing the OGG1-GFP fusion protein (lower panel). Plots below the gel images report fraction of DNA in the open circular form (oc) as a function of the extract concentration. Nicking activities towards analogous constructs containing F at the same positions in the “R” and “Y” strands are shown for comparison (purple bars in the plots). Representative results of one of four reproducible experiments. (For interpretation of the references to colour in this figure legend, the reader is referred to the Web version of this article.)

Based on the observation that 8-oxodG in the CpG dinucleotide of the GC box can influence the gene expression either in the OGG1-dependent or OGG1-independent manner (as defined by the DNA strand carrying the modification), we questioned whether the OGG1 DNA glycosylase activity has a preference for one of the DNA strands of the GC box element. To address this question, we compared the cleavage rates of constructs containing 8-oxodG in either strand (the purine-versus pyrimidine-rich) of the GC box by performing in vitro incision assays with human OGG1 (hOGG1) DNA glycosylase. Taking into account the reported low processivity of pure OGG1 at the post-excision step and its stimulation by APE1 [[56], [57], [58]], we analysed the incision activities in the presence and in the absence of APE1. Under both conditions, we titrated pure hOGG1 to identify a concentration range, which generates incomplete but measurable conversion of covalently closed circular (cc) DNA containing a single 8-oxodG residue into the open circular form (data not shown). Under the established conditions, we directly compared the incision activities towards 8-oxodG placed in the purine-rich versus the pyrimidine-rich strand by quantifying the incision rates of plasmid DNA at equivalent enzyme concentrations (Fig. 3A). The results showed that, under both conditions, single synthetic 8-oxodG at the central CpG dinucleotide of the GC box was cleaved much more efficiently, when located in the pyrimidine-rich strand. Thus, in the absence of APE1, 0.02 U hOGG1 induced conversion of the vector DNA into the open circular (“oc”) form at the rates of 52.1 ± 0.8% for 8-oxodG placed in the pyrimidine-rich DNA strand but only 32.3 ± 1.0% in the purine-rich strand. Importantly, only a very minor degree of cleavage was observed in control constructs containing dG (<7% increase of the “oc” form in addition to 11% already present in the absence of hOGG1), indicating that the levels of intrinsic OGG1-sensitive base modifications at random positions in the vector DNA were negligible (Fig. 3A, upper panel). Thereby, the observed differences in cleavage efficiencies between the pGCbox-W and pGCbox-C constructs should be entirely assigned to specific 8-oxodG sites within the GC box.

In the absence of APE1, the incision rates represent combined DNA glycosylase and beta-lyase activities of OGG1. As expected, the rates of strand cleavage at the 8-oxodG sites were enhanced by addition of APE1, resulting in almost full conversion of both 8-oxodG-containing constructs into the oc form at unchanged hOGG1 concentration. Nonetheless, also in the presence of APE1 (Fig. 3A, upper gel, last lanes in the “8oG” series), the cleavage was more complete, when 8-oxodG was present in the pyrimidine-rich strand of the GC box sequence, in agreement with the preferential OGG1 activity towards 8-oxodG in this strand. We thus conclude that, also in the presence of APE1, differential activities towards the 8-oxodG substrates should be attributed entirely to OGG1.

As cleavage rates in the presence of APE1 and 0.02 U hOGG1 were too high for a quantitative comparison between the substrates containing 8-oxodG in different DNA strands of the GC box, we repeated the cleavage assay under a five times lower concentration of hOGG1 (Fig. 3A, lower gel). Using 0.004 U of hOGG1, the preferential cleavage of 8-oxodG in the pyrimidine-rich strand was clearly confirmed: the fraction of DNA in the “oc”-form increased by 66.4 ± 0.4%. With 8-oxodG in the purine-rich strand, the corresponding increase was only 34.4 ± 0.3%. Besides the incision at the specific lesion sites, some background nicking of “dG” constructs incubated with OGG1 in the presence of APE1 was also taking place (Fig. 3A). This was expected, because all plasmid DNA preparations inherently contain some AP-sites arisen by spontaneous depurination. Notably, the control “dG” construct with substituted pyrimidine-rich strand of the GC box did not display an enhanced cleavage rate at these unspecific sites, compared to the counterpart in which the purine-rich strand was substituted (a 10.5% increase in the “oc” form versus 12.9%, respectively). Thus, background damage could not account for the preferential incision of the 8-oxodG substrate containing the modification in the pyrimidine-rich as compared to the purine-rich strand of the GC box. In summary, the comparison of the hOGG1 activities in the presence or absence of APE1 indicated that incision rates of 8-oxodG were enhanced by APE1 by a factor greater than five in both sequence contexts. Still, APE1 had no effect on the strand preference of the incision, which strongly suggests that the observed specificity for the pyrimidine-rich strand of GC box should be solely attributed to OGG1.

To evaluate incision efficiencies at 8-oxodG in both the purine-rich and the pyrimidine-rich DNA strands under the physiological stoichiometry of the BER components, we next performed cleavage assays with cell extracts (Fig. 3B). Since OGG1 is rate-limiting for the BER of 8-oxodG in human cells [57,59,60], in parallel, we used protein extracts obtained from both HeLa cells and the derived cell line overexpressing a functional OGG1-GFP fusion protein [47]. In line with relatively low endogenous OGG1 glycosylase activities reported previously [46,59], cleavage activity towards the 8-oxodG containing constructs was barely detectable in extracts obtained from the wild-type HeLa cells (Fig. 3B, upper gel), but was readily observed under the OGG1 overexpression conditions (Fig. 3B, lower gel). Once again, as in the case of purified proteins, the pyrimidine-rich strand was cleaved more efficiently than the purine-rich strand. Based on the cell extract dilution factor required for an equivalent degree of conversion into the “oc” form, the cleavage rates differed between the examined constructs by more than 2-fold. We attribute this difference specifically to 8-oxodG, since the corresponding dG control constructs were incised at very minor rates and without any difference between the pyrimidine-rich and purine-rich DNA strands.

Keeping in mind that the strand incision product is a result of two consecutive reactions catalysed by OGG1 and APE1, we wanted do differentiate between these two activities. We, therefore, constructed also substrates containing synthetic AP lesions (F) at the same positions as 8-oxodG in the purine- and pyrimidine-rich DNA strands (Supplementary Fig. S3). As expected, constructs containing the F lesion were cleaved, regardless of the OGG1 activity present in the cell extracts (Fig. 3B, last lanes in both gels). The incision efficiencies at F were higher than at 8-oxodG, regardless of the DNA strand and of the OGG1 status of the cell extract, in agreement with the expectation that the rate-limiting step of the entire 8-oxodG incision reaction was the base removal by OGG1. Thus, in the case of Hela cells extracts, the percentage fractions of nicked DNA related for the dG/8-oxodG/F constructs as 26.4/26.1/66.2 in the purine-rich strand and 27.5/32.8/94.7 in the pyrimidine-rich strand, based on the results of the experiment shown in Fig. 3B. For Hela-OGG1-overexpressing extracts, the corresponding values were 22.1/50.1/69.5 and 23.4/90.0/100.0.

Taken together, we conclude that BER strand cleavage activity by OGG1 (alone or in concert with APE1) towards 8-oxodG in the GC box sequence has a clear preference to the pyrimidine-rich over the purine-rich DNA strand. Thus, the strand specific excision of 8-oxoGua correlates with the downregulation of the gene expression observed in the reporter assay (Fig. 2), which was significant only when 8-oxodG was present in the pyrimidine-rich DNA strand. Taking into account the OGG1-dependency of the gene repression, the results strongly suggest a causal connection between the base excision and the negative outcome for the promoter activity.

### 3.4 APE1 may contribute to the strand incision preference at the 8-oxodG sites

**Fig. 4:**
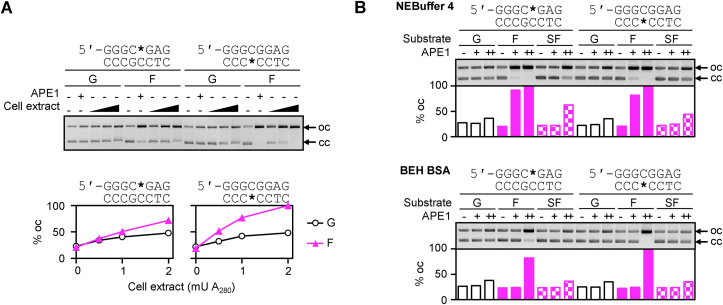
Incision efficiencies of apurinic lesions in opposite DNA strands of the GC box. (A) Incision of constructs containing dG or the tetrahydrofuran AP lesion (F) at the indicated positions (*) by pure APE1 or the extract of HeLa cells overexpressing the OGG1-GFP fusion protein (the same as analysed in Fig. 3). Identical results were obtained with extracts of the parental HeLa cells (Supplementary Fig. 4). (B) Incision by 0.005 units (+) and 1 unit (++) of pure APE1 in the magnesium-containing NEBuffer 4 (upper panel) and the magnesium-free BEH-BSA buffer (lower panel). Constructs with AP lesions (F) were the same as in (A). APE1-resistant constructs containing tetrahydrofuran lesions with phosphorothioate 5′-linkages (SF) were incubated in parallel. (A) and (B) show representative results of three reproducible experiments.

Irrespective of the clearly different hOGG1 activities towards 8-oxodG in opposite strands of the GC box, we noticed that the F lesions were also incised with different efficiencies, depending on the DNA strand. At the highest cell extract concentration, this corresponded to 66.2% and 94.7% DNA in the “oc” form for constructs containing F in the purine-rich and the pyrimidine-rich DNA strand, respectively (Fig. 3B, lower gel). To quantitatively compare the cleavage rates of F in the different DNA strands, we performed cleavage reactions under limited incision conditions with serial dilutions of the cell extract (Fig. 4A and Supplementary Fig. S4). The results showed that, similarly to 8-oxodG, F was also incised more efficiently when located in the pyrimidine-rich strand. However, it is unclear whether the observed incision preferences have a potential biological relevance, because these cleavage reactions were performed in the presence of EDTA to prevent DNA degradation by nucleases present in the cell extracts. Unfortunately, quantitative cleavage assays with cell extracts could not be performed in the magnesium concentration range, which would be optimal for APE1, because of the high rates of non-specific cleavage by other nucleases (data not shown). To solve this problem, we next incubated the F substrates with purified human APE1 both under the optimal magnesium concentration (NEBuffer4) and in the presence of EDTA as a chelating agent (BEH-BSA) (Fig. 4B). In agreement with previous reports [61,62], we observed a strong (approximately 200-fold) stimulation of specific nicking of the F substrates by human APE1 in the presence of magnesium. Notably, the preferential cleavage of F in the pyrimidine-rich strand was no longer observed under the optimal APE1 cleavage conditions.

The existence of two distinct endonuclease activities, depending on the substrate structure and the availability of magnesium is a peculiar property of APE1 [[62], [63], [64]]. The canonical endonuclease activity during BER of AP lesions requires magnesium ions in a millimolar concentration range with an optimum between 2 and 10 mM, whereas at submillimolar concentrations of magnesium, a so-called nucleotide incision repair (NIR) activity dominates [65]. Based on the preferences of strand scission observed under cell-free conditions, we cannot rigorously exclude that NIR activity could contribute to an asymmetric processing of both 8-oxodG and the derived abasic lesions in the intracellular environment. Nevertheless, the results indicate that APE1 activity in cell extracts greatly exceeds the availability of OGG1. Thereby, the strand selectivity of 8-oxodG processing should originate primarily from OGG1 as the rate limiting factor.

### 3.5 AP lesions in the GC box mildly affect the promoter activity, but cause a strong repression if incised on the 5′-side

The results so far indicated that the outcomes of 8-oxodG for the GC box-driven gene expression are largely mediated by OGG1; however they did not address the role of APE1 in transcriptional repression or activation. To understand the impact of the downstream lesion processing steps on the promoter activity, we constructed expression constructs containing F, as a chemically stable synthetic AP lesion, at the positions formerly used to analyse the effects of 8-oxodG. F is a close structural analogue of natural AP lesions processed by APE1 and an excellent APE1 substrate [64,66]. For each DNA strand, we constructed two versions of the F lesion: one with canonical phosphodiester 5′ and 3′ linkages and another with APE1-resistant phosphorothioate linkage on the 5′ (further referred to as SF) [67]. Both (F and SF) lesions were very efficiently incorporated into both positions in the GC box (Supplementary Fig. S3). As expected, the F lesion was efficiently cleaved by APE1, whereas the incision at the SF lesion was inhibited by a factor of >200 (Fig. 4B). This made SF an ideal model of a repair-resistant AP lesion, suited for investigation of effects on the GC box activity in cells. Furthermore, by comparing SF and F lesions, we hoped to elucidate functional outcomes of the incisions by APE1 in each DNA strand.

**Fig. 5:**
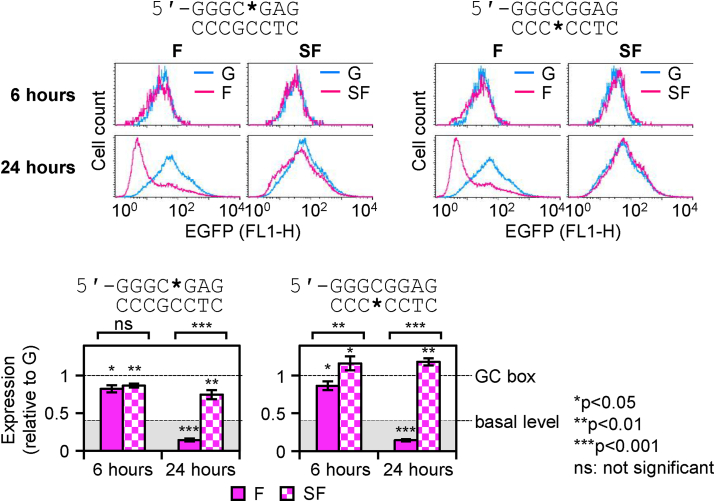
Impacts of apurinic lesions in opposite DNA strands at the central CpG dinucleotide of the GC box promoter on the gene expression. EGFP expression in HeLa cells 6 and 24 h after transfection with constructs containing synthetic AP lesions (F or SF) at the indicated positions (*). Note the left shifting of the F peaks in the fluorescence distribution plots and its attenuation by the phosphorothioate 5′-linkage (SF). Bar charts (underneath) show quantification of expression of constructs containing F or SF, relative to the reference constructs containing dG at the same positions (mean ± SD, n = 5). Basal expression level is inferred from the reporter vector lacking a GC box (Supplementary Fig. 1). Statistical significance (P-values calculated by Student's heteroscedastic, two tailed *t*-test) for differences between the given type of AP lesion and dG are indicated above the individual columns, whereas differences between different types of AP lesions (F versus SF) are reported with brackets above the charts.

Analyses of EGFP expression in transfected HeLa cells showed that APE-resistant SF lesion in the purine-rich strand of the GC box sequence led to a mild decline of the promoter activity (86.8 ± 2.5% and 74.8 ± 5.8% residual expression, 6 and 24 h post transfection), which was statistically significant at both time points (Fig. 5). Strikingly, SF in the pyrimidine-rich strand caused an opposite effect, with a significant enhancement of the gene expression documented at both time points (116.3 ± 9.3% at 6 h and 118.4 ± 4.8% at 24 h). Considering that the minimal GC box promoter offers a rather narrow, approximately two-fold, dynamic range of transcriptional activation in relation to the basal level of transcription (Supplementary Fig. S1), we regard the effects of AP lesions in both strands as potentially biologically meaningful. Unlike the APE1-resistant SF lesion, the F lesion induced a strong decline of the gene expression between 6 and 24 h, regardless of the DNA strand (Fig. 5). Thereby, the outcome of the reparable AP lesion resembled the effect previously observed for the 8-oxodG situated in the pyrimidine-rich strand of the GC box (Fig. 2), with the difference that the AP lesion induced gene silencing irrespectively of the DNA strand. Thus, by 24 h post transfection, the residual EGFP expression of constructs containing F in the purine- and the pyrimidine-rich strand declined to the levels of 14.6 ± 1.6% and 14.1 ± 1.4%, as compared to the respective reference constructs containing dG. Noteworthy, these levels lie distinctly below the basal expression level of a GC box-less reporter. We, therefore, conclude that incision of AP lesions by APE1 triggers gene silencing, in a similar mode of action as previously described for lesions situated in transcribed region of genes controlled by unrelated promoters [49]. As the incision by APE1 is efficient in both DNA strands of the GC box (Fig. 4B), AP lesions in different DNA strands inflict similar transcriptional silencing responses. By contrast, the strand-specific induction of gene silencing in the case of 8-oxodG (Fig. 1, Fig. 2) can now be attributed to OGG1 and not to APE1, in coherence with the strand selectivity of incision observed for that type of DNA lesion (Fig. 3).

### 3.6 8-oxodG residues at three conserved positions in the purine-rich strand all negatively impact the GC box activity, but in subtly different ways

**Fig. 6:**
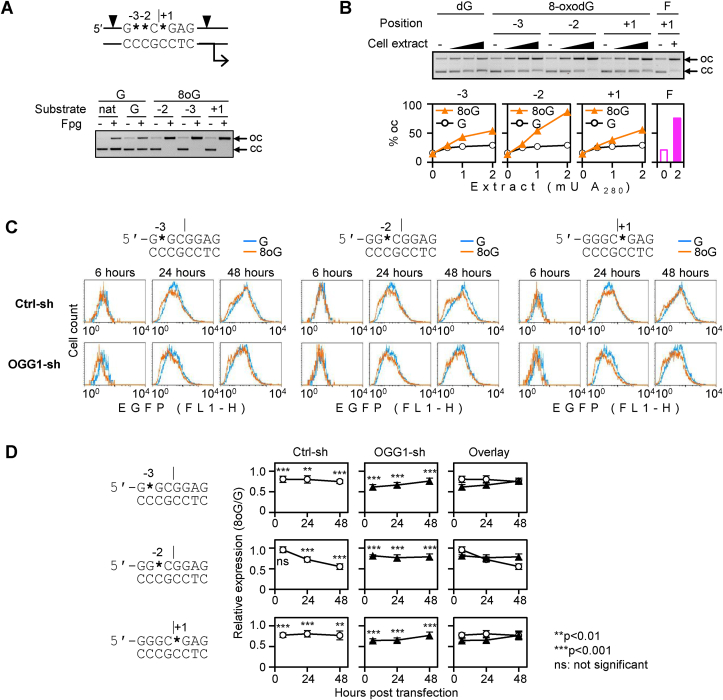
Generation of EGFP expression constructs containing 8-oxodG at three selected positions in the purine-rich strand of the GC box, the impacts of 8-oxodG on the gene expression, and the effects of OGG1. (A) Generation of EGFP expression constructs containing 8-oxodG at three selected positions in the purine-rich strand of the GC box and verification of the presence of 8-oxodG by the incision with Fpg. Wildcards (*) indicate positions of synthetic 8-oxodG residues in the purine-rich strand, numbered with respect to the axis of the central CpG dinucleotide. Black arrowheads show Nb.BsrDI nicking sites, the broken arrow indicates the transcription start site. (B) Quantification of incision of 8-oxoG at three positions in the purine-rich strand of the GC box. The constructs were incubated with increasing amounts of HeLa OGG1-GFP cell extract (as in Fig. 3B). Observe different efficiencies of incision at 8-oxodG in different positions. An independent construct containing an AP lesion (F) at position +1 was used as an indicator of the APE1 activity in the extract. The incision at 8-oxodG was undetectable in the extracts of the parental HeLa cells (Supplementary Fig. S5). (C) EGFP expression time course in the OGG1 proficient (Ctrl-sh) and in the OGG1 knock down (OGG1-sh) HeLa-derived cell lines. Fluorescence distribution plots of a representative experiment. (D) Quantification of EGFP expression relative to the reference “dG” construct (mean ± SD, n = 7) in the congenic (“Ctrl-sh” and “OGG1-sh”) cell lines. P-values: Student's heteroscedastic, two tailed *t*-test.

Considering very different outcomes of 8-oxodG residues in different DNA strands of the GC box, it was interesting to analyse the impacts of 8-oxodG located at various positions in the purine-rich (“R”) strand. Within the chosen GC box consensus sequence, guanines are present at positions R-4, R-3, R-2, R+1, R+2, and R+4 (with G of the central CpG dinucleotide indexed as R+1). In addition to position R+1 analysed in the previous experiments, we chose positions R-2 and R-3 as the most conserved in the mammalian GC box consensus sequence [52] and generated EGFP expression constructs containing 8-oxodG at positions R-3, R-2, and R+1 (Fig. 6A and Supplementary Fig. S2). In all three types of the reporter constructs, 8-oxodG was fully cleaved by Fpg, indicating that the GC box was correctly folded. Next, we analysed the incision efficiencies at different positions by incubating covalently closed plasmid DNA with extracts of OGG1-overexpressing HeLa cells (Fig. 6B). The incision efficiency at position R-3 was as low as at the reference position R+1, whereas at position R-2 it was somewhat (<2-fold) higher (Fig. 6B). We thereby conclude that the processing efficiencies of 8-oxodG by BER differ slightly along the purine-rich strand of the GC box. When incision reactions were performed with purified hOGG1 in the presence of APE1, 8-oxodG at R-2 was again cleaved with the highest efficiency among the three analysed positions (Supplementary Fig. S5). In summary, based on the results shown in Fig. 3, Fig. 6B and on other available data (not shown), we ranked the incision preferences of 8-oxodG at positions analysed in this study as Y-1>R-2>R-3≈R+1.

As previous results suggested that transcriptional silencing rates of GC box constructs containing 8-oxodG in the opposite DNA strands are dependent on the incision efficiencies, we next assessed whether the subtle differences between the analysed positions in the purine-rich strand lead to different outcomes for the promoter activity. In the OGG1-depleted cell line, 8-oxodG at any of the three positions caused a significant decline of the EGFP expression, which showed no or little (in the case of position R-2) change in the magnitude throughout the time period between 6 and 48 h (Fig. 6C and D). This indicates that oxidised guanines at the most conserved positions of the consensus GC box motif directly interfere with the transcriptional function when the repair is absent or inefficient. For 8-oxodG at positions R-3 and R+1, the outcomes in the matched OGG1-proficient cell lines were the same, or very similar, as in the OGG1-knockdown cells (Fig. 6C and D and Supplementary Fig. S6). In contrast, 8-oxodG at position R-2 displayed a steady decline of the gene expression in OGG1-proficient cell lines (both the empty vector control “Ctrl-sh” and the maternal HeLa cells) between 6, 24 and 48 h post transfection. Although modest in its magnitude, this effect was highly reproducible and significant. Thereby, BER initiation by OGG1 led to a time-dependent decrease of transcriptional activity of the construct containing 8-oxodG at position R-2. As this did not happen when 8-oxodG was present at position R-3 or R+1, it is tempting to suggest that the incision might simply not take place at these positions. Even though the incision efficiencies cannot be measured in the intracellular environment, such an explanation would be in accordance with the observed preferences of BER incision at the analysed positions (R-2>R-3≈R+1). In all, we conclude that unrepaired 8-oxodG at all analysed positions in the purine-rich strand of the GC box directly interferes with the transcriptional activation. In addition, promoter activity is affected by BER to varying degrees at different positions, as defined by the sequence preferences of OGG1.

## 4 Discussion

By placing synthetic 8-oxodG into four specific positions within an artificial elementary promoter, constituted of a standalone GC box, we discovered a remarkable heterogeneity in the impacts of this common oxidatively generated DNA modification on the GC box activity. By modulation of the two initial steps of BER (knockdown of OGG1 and the use of APE-resistant synthetic AP lesion), we revealed two different modes of action, which explain the observed functional outcomes of 8-oxodG. The first mechanism, applicable to 8-oxodG in all analysed positions in the purine-rich strand, is the direct impairment of the transcriptional activation. This mechanism is BER-independent, as it is not influenced by the OGG1 expression levels, and the negative effects of 8-oxodG are observable immediately from the moment the reporter gene expression sets up (Fig. 2, Fig. 6). The weakening of the GC box activity by 8-oxodG at positions −3, −2 and +1 in the purine-rich strand is in line with diminished Sp1 transcription factor binding, reported previously for 8-oxodG in these positions [33,35]. By contrast, 8-oxodG in the pyrimidine-rich DNA strand did not cause such a direct impairment of the transcriptional activity or did it to a much milder degree than the modifications in the opposite DNA strand (Fig. 2). Even though 8-oxodG in the corresponding position also reduced the Sp1 binding [35] and despite the fact that this C:G base pair is the best conserved within the GC box consensus sequence [52], our results suggest that oxidation of guanine in the pyrimidine-rich strand is relatively well tolerated for the sustainment of the GC box activity in the cellular context.

The second mechanism revealed by our results is the OGG1-dependent transcriptional repression initiated by the excision of 8-oxodG. In contrast to the direct effects of 8-oxodG described above, its onset was delayed and the impact on the gene expression this time was the strongest in the case of 8-oxodG in the pyrimidine-rich strand. By using AP lesions introduced into various gene regulatory regions, we and others have previously reported that it was their cleavage by APE1 that induced transcriptional silencing of the affected genes [36,41,44]. The mechanism downstream from the APE1 step is likely to be independent from the promoter type, since it involves reorganisation of the chromatin structure over the range of hundreds of base pairs and apparently does not require that the nick is generated within a particular regulatory element [49,51]. Nonetheless, for 8-oxoGua emerged in a peculiar sequence element, the specificity can be provided already at the OGG1 step. Thus, within the GC box sequence, the magnitude of the observed gene silencing was clearly governed by the excision preferences: only 8-oxoGua at positions well excised by OGG1 (R-2 in the purine-rich DNA strand of the GC box and Y-1 in the pyrimidine-rich strand) caused transcriptional silencing (Fig. 2, Fig. 3, Fig. 6). By contrast, at positions R-3 or R+1, 8-oxoGua was poorly excised under cell-free conditions and, accordingly, induced no additional gene silencing in cells, but rather sustained the GC box activity at slightly decreased yet steady levels (Fig. 6). By extrapolation, relative resistance to OGG1 might lead to prolonged persistence of 8-oxoGua in specific sequence contexts in the genome, which would be essential to enable its function as a stable regulatory mark, proposed previously by Burrows and co-workers [39]. In support of this notion, nucleotide-resolution mapping over the yeast genome revealed a pronounced enrichment of 8-oxodG at the 5′-position of GpG dinucleotides [11]. Although originally attributed to irregular sensitivities to oxidation, this could as well result from the disfavoured processing by OGG1, as this very motif was depleted from the mutation signature obtained by the OGG1 gene disruption in human cells [68]. The impairment of hOGG1-catalysed 8-oxoGua excision was reported previously in two sequence contexts containing G as the nearest neighbour on the 3′ [46] and is fully concordant with findings that excision is disfavoured at positions R-3 or R+1 of the GC-box, both of which are in the 8-oxodGpG dinucleotides (present study).

At none of the four analysed positions did we observe enhancement of the GC box activity by 8-oxodG (Fig. 2, Fig. 6). This is overall in agreement with previous reports that activation of several GC-rich promoters by 8-oxoG required folding into G-quadruplex (G4) structures [37,39] and that the effects of 8-oxodG reversed from activation to repression when potential G4 forming sequences were eliminated from the promoters [41]. Of note, although the promoter sequence used in our work does not contain any longer-range GC-rich elements, which would be prone to folding into a non-B DNA structure, we nevertheless observed stimulation of GC box activity by uncleavable AP lesion (SF) in the pyrimidine-rich strand (Fig. 5). To our knowledge, previous studies only addressed the effects of AP lesions in the pyrimidine-rich DNA strands of GC-rich regulatory elements (some of which contained putative GC box sequences), leading to a model that DNA folding into non-canonical structures prevents cleavage by APE1 and that non-processive binding of APE1 enhances the activation of transcription [36,37,39]. Our new data indicates that the presence of an AP lesion can have opposite effects on transcriptional activity already at the level of a standalone GC box, depending on the DNA strand. Furthermore, comparison between F and SF lesions, demonstrates the key role of APE1 activity in defining whether the final outcome would be transcriptional activation or repression (Fig. 5).

Although the cleavage of AP sites uniformly led to transcriptional repression under the chosen experimental conditions in our cell model, in the future it would be interesting to explore the regulation mechanisms of APE1 activity as a potential switch in the control of gene expression. Human APE1 is subject to numerous posttranslational modifications, whose roles in the regulation of its enzymatic activity still have to be deciphered. Moreover, regardless of its endonuclease function, APE interacts with numerous transcription factors and regulates them through a redox activity. Taking into consideration the great complexity of biological functions and regulation mechanisms of APE1 reviewed elsewhere [[69], [70], [71], [72], [73]], its role in gene activation or repression might be regulated at multiple levels of processing of DNA base modifications and abasic sites, depending on the cell type and, perhaps, exogenous signals or stressors. Intriguingly, our results provide evidence that strand selectivity of the APE1-catalysed cleavage can be modulated by the availability of magnesium (Fig. 4). This is probably because the alternative catalytic modes of APE1 are differently regulated by magnesium concentration [62,66]. The availability of magnesium largely defines the structural and dynamic properties of DNA [74,75], which in turn impact the APE1 binding and activity in a sequence-dependent fashion [64]. It remains to be established whether the mentioned mechanisms may contribute to the regulation of transcription by modulating the cleavage of AP lesions in gene promoters.

In summary, our systematic investigation of position-specific effects of 8-oxodG on the activity of a GC-rich regulatory element revealed a notable complexity of the regulation of transcriptional responses even in the simplest promoter consisting of a single GC box. The results show that, in addition to direct inhibition of the GC box activity by 8-oxodG, the key BER components OGG1 and APE1 mediate the regulation of gene transcription in position-specific manners. The mechanisms described here do not require DNA folding into a G4 structure, yet they are likely to contribute to more elaborated transcriptional regulation by operating at elementary units of more complex promoters described in the literature [25,26,37,39,43]. Taking into account cooperativity of action of different transcription factors, involvement of BER proteins into these interactions, and capacities of OGG1 and APE1 as well as their substrates to regulate DNA folding into non-conventional structures [23,29,37,39,45], the understanding how these mechanisms integrate during regulation of complex promoters in the genome is an exciting and challenging task for future research.

## Funding

This work was supported by the 10.13039/501100001659Deutsche Forschungsgemeinschaft (DFG, German Research Foundation) [Project-ID 393547839 - SFB 1361; grant numbers INST 247/926, KH263/1, KH263/2 and Heisenberg grant KH263/5 to A.K.]. Funding for open access charge: DFG, German Research Foundation.

## Declaration of competing interest

The authors declare that they have no known competing financial interests or personal relationships that could have appeared to influence the work reported in this paper.
