# Supplementary material for: Regulation of GC box activity by 8-oxoguanine
Source: Redox Biol. 2021 Apr 30;43:101997. doi: 10.1016/j.redox.2021.101997 (PMC8120935; doi:10.1016/j.redox.2021.101997)
Supplement: Multimedia component 1 [file mmc1.pdf]

Regulation of GC box activity by 8-oxoguanine

Nadine Müller and Andriy Khobta

Supplementary information:

- Supplementary Figure S1. Construction and functional characterization of reporter vectors expressing the EGFP gene under the control of a standalone GC box.

- Supplementary Figure S2. Generation of reporter constructs containing 8-oxo-dG at the indicated positions within the GC box.

- Supplementary Figure S3. Generation of reporter constructs containing apurinic lesions at the central CpG dinucleotide of the GCbox.

- Supplementary Figure S4. Incision analysis of vectors containing single abasic sites in the central CG dinucleotide of the GC box by HeLa cell extract.

- Supplementary Figure S5. Incision of 8oG at three positions in the purine rich strand of the GC box by OGG1 and by HeLa cell extract.

- Supplementary Figure S6. Impacts of 8-oxodG at three selected positions in the purine-rich strand on the GC box activity.

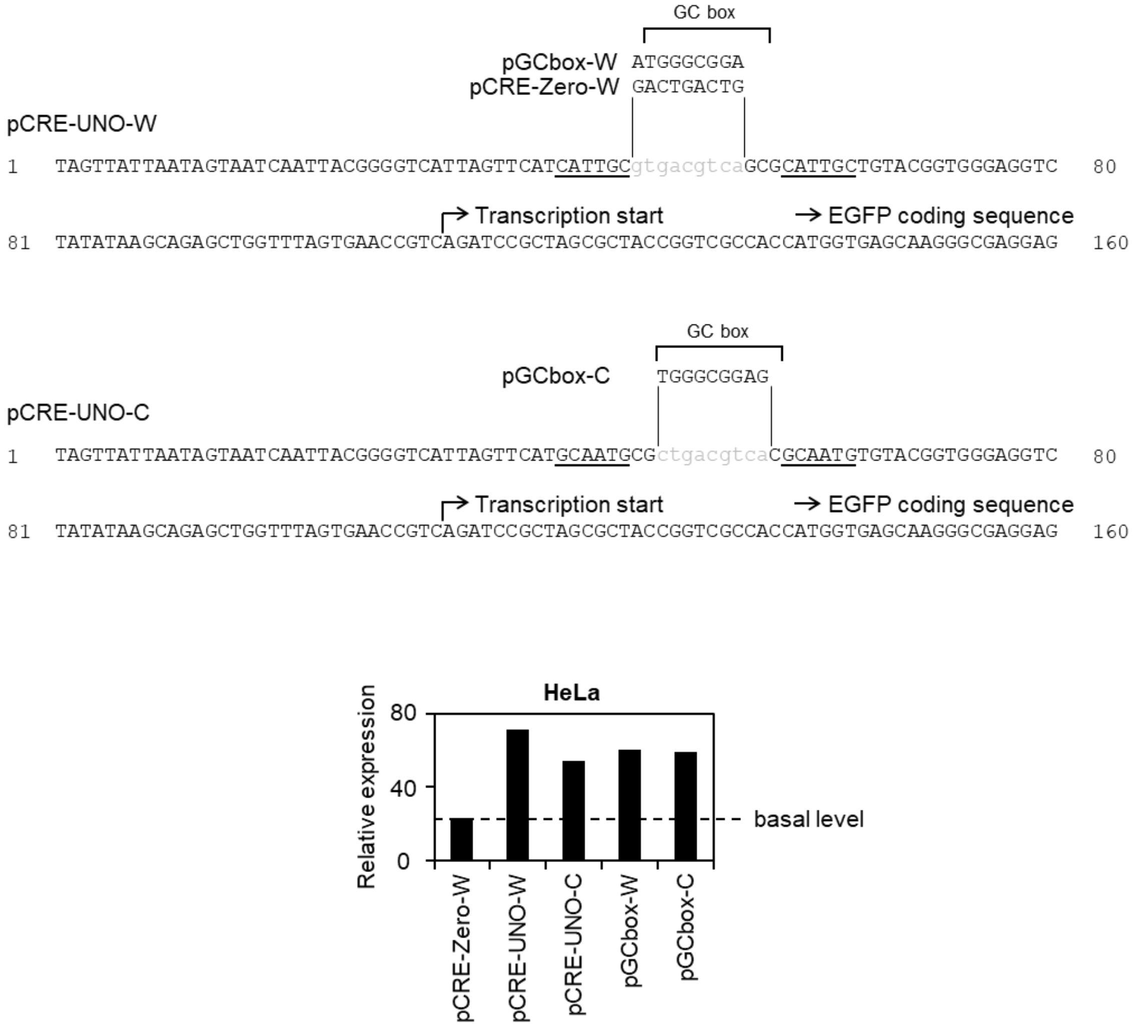

Supplementary Figure S1. Construction and functional characterization of reporter vectors expressing the EGFP gene under the control of a standalone GC box.

The vectors pCRE-UNO-W and pCRE-UNO-C were described previously [44]. The first 160 nucleotides of the original vector sequences are shown, including the transcription start site (broken arrow), the begin of the EGFP coding sequence (straight arrow) and the fragments substituted to generate the pGCbox-W, pGCbox-C and pCRE-Zero-W vectors (insets with vector names and the respective nucleotide sequences). The newly obtained pGCbox-W and pGCbox-C vectors contain a common GC box consensus sequence 5′-TGGGCGGAGC-3′ (bracket above the sequence), which is flanked on both sides by the Nb.BsrDI sites (underlined) retained from their precursor vectors. Note the same direction of the GC box sequence in both vectors as well as inverted orientations of the Nb.BsrDI sites. The pCRE-Zero-W vector was constructed in an analogous way as pGCbox-W, but the sequence between the BsrDI sites was deprived of any acknowledged transcription factor-binding motif. Bar chart below shows promoter activities of the described vectors, as measured by quantitative EGFP expression analyses in Hela cells 24 hours after transfection.

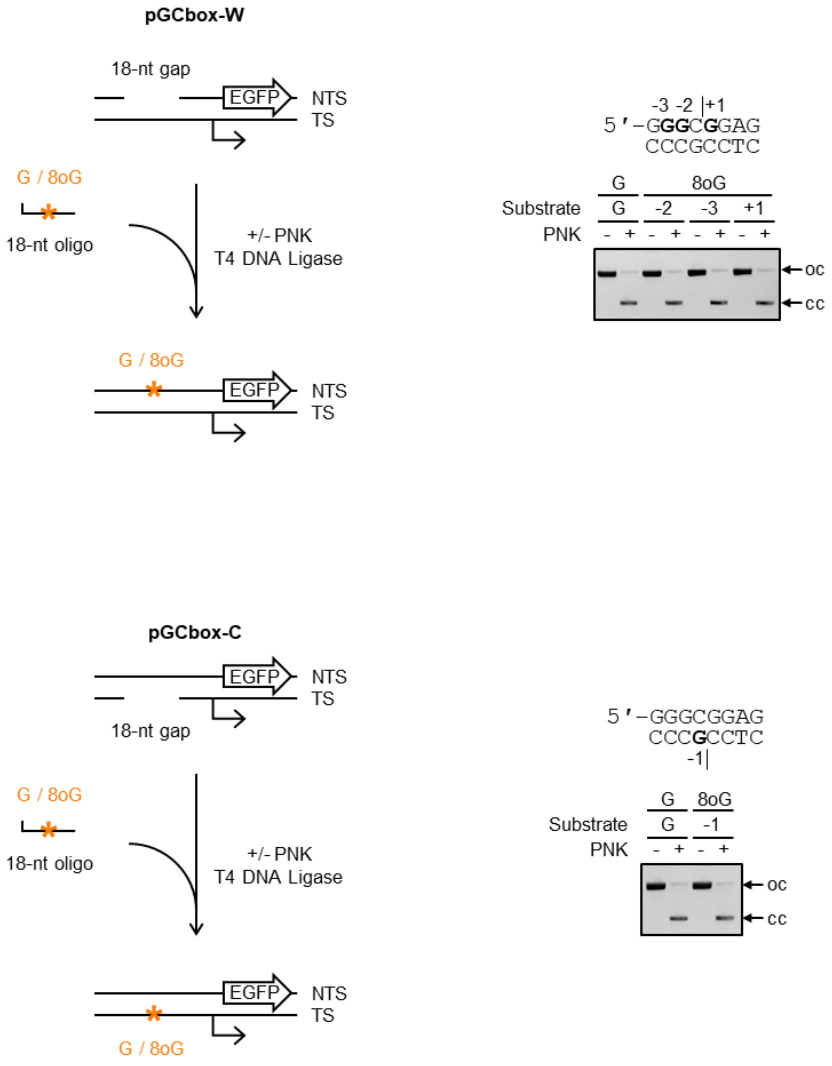

Supplementary Figure S2. Generation of reporter constructs containing 8-oxodG at the indicated positions within the GC box.

Schemes of the pGCbox-W and pGCbox-C constructs indicating the position of the guanine substituted for 8-oxodG (G/8oG), transcription start (broken arrow), transcribed and non-transcribed DNA strands (TS, NTS) and the EGFP coding sequence. Single-stranded gaps in the promoter sequences were generated by double nicking with Nb.BsrDI and depletion of the excised strand, as described in Materials and Methods. Matched synthetic oligonucleotides containing dG or 8-oxodG at chosen positions were seamlessly ligated into the gaps, as verified by generation of the covalently closed form (cc). In parallel, the completeness of swapping of the native strand for the synthetic oligonucleotides was verified by inhibition of ligation in the absence of polynucleotide kinase (PNK). The presence of 8-oxodG and correct folding of the respective constructs was further validated by incision with Fpg DNA glycosylase (as seen in Figure 1B)

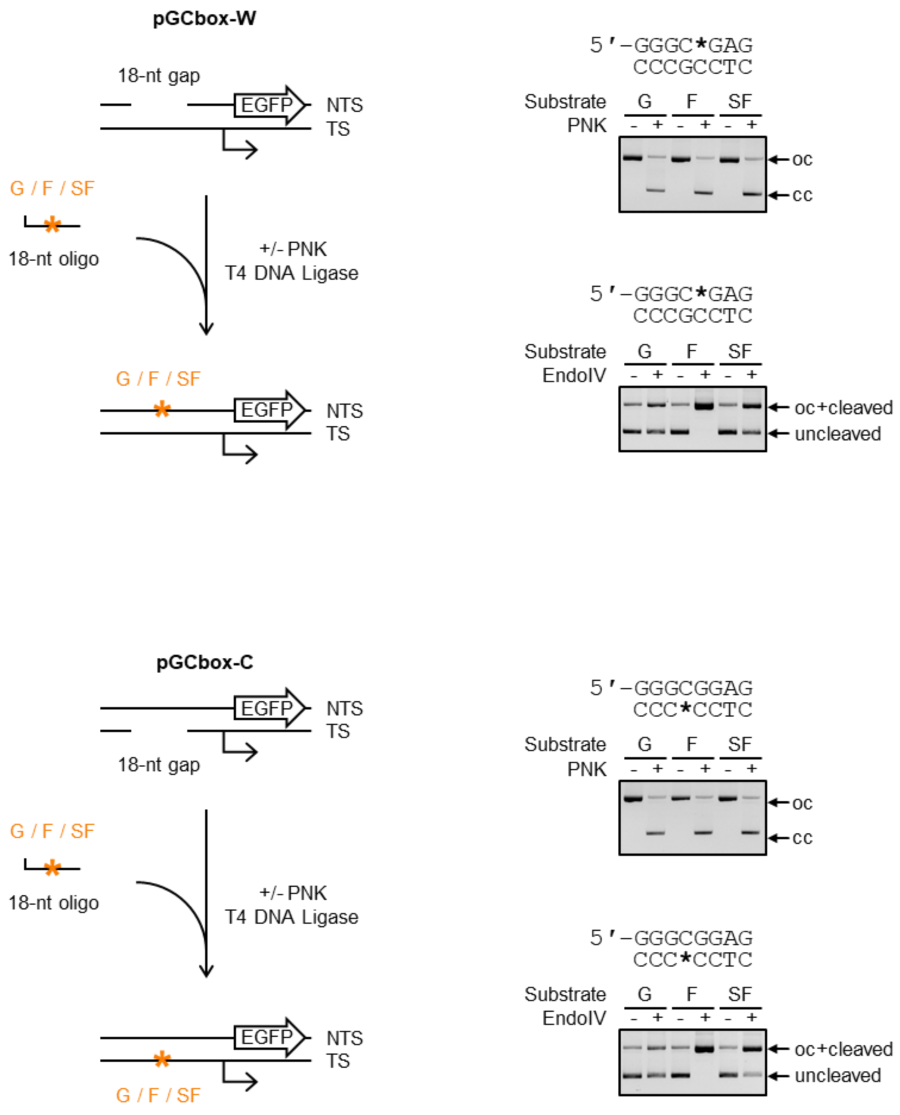

Supplementary Figure S3. Generation of reporter constructs containing apurinic lesions at the central CpG dinucleotide of the GC box.

Scheme of the incorporation of synthetic oligonucleotides containing dG, F or SF into the gapped pGCbox-W and pGCbox-C vectors. Correct incorporation of the synthetic strands into the gap was verified by ligation in the presence or absence of PNK. The presence of F was additionally validated by incision with E. coli Endonuclease IV (Endo IV).

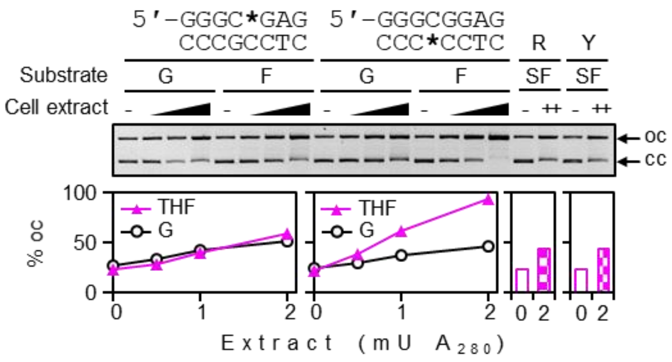

Supplementary Figure S4. Incision analysis of vectors containing single abasic sites in the central CpG dinucleotide of the GC box by HeLa cell extract.

Constructs containing synthetic AP lesions (F) at the central CpG dinucleotide or the respective dG controls were incubated with increasing amounts of cell extracts, as described in Materials and Methods. Constructs containing uncleavable SF lesions in either the purine-rich (R) or the pyrimidine-rich strand (Y) were incubated in parallel with the highest extract concentrations. Graphs show quantification of the open circular fraction (oc) in the agarose gel.

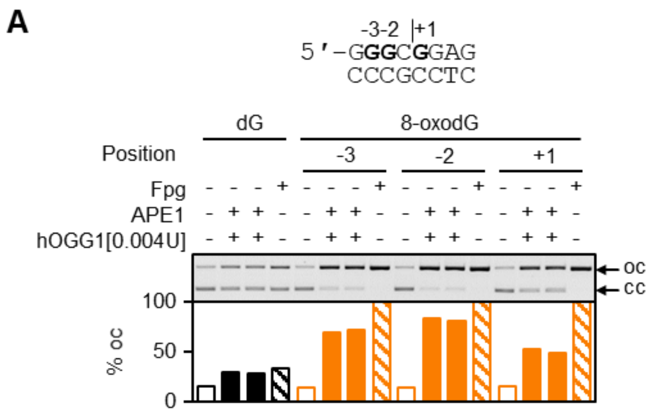

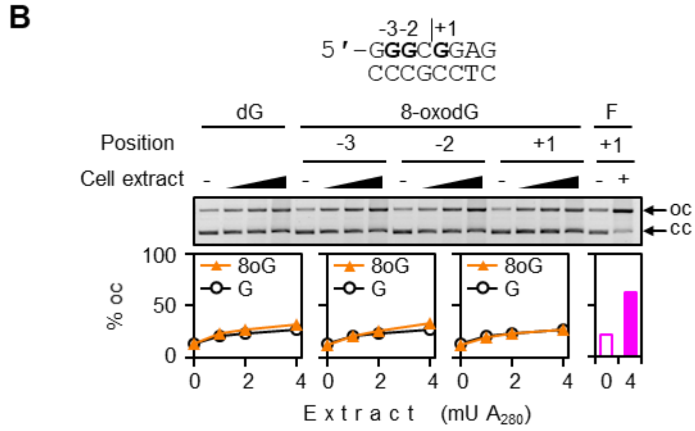

Supplementary Figure S5. Incision of 8-oxodG at three positions in the purine-rich strand of the GC box by OGG1 and HeLa cell extract.

Constructs containing 8-oxodG at each of the three indicated positions (*) in the purine rich strand of the GC box were incised by incubation with purified human OGG1 in the presence of 1 U APE1/100 ng plasmid DNA (A) or with increasing amounts of HeLa cell extract (B). The cleavage was quantified by quantification of the open circular construct fraction in the agarose gels. Incision of the control construct containing a single AP lesion (F) at position +1 indicates that APE1 was not rate limiting under the given conditions.

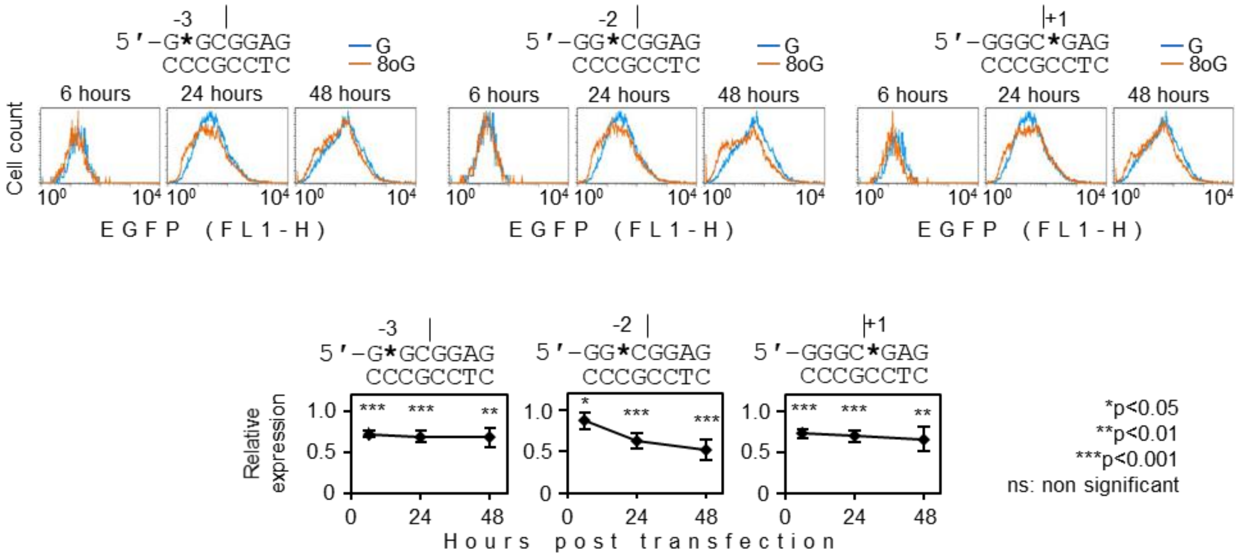

Supplementary Figure S6. Impacts of 8-oxodG at three selected positions in the purine-rich strand on the GC box activity.

EGFP expression time course in HeLa cells transfected with constructs containing single 8-oxodG in the indicated positions (*). Representative fluorescence distribution plots and quantification of EGFP expression (mean ± SD, n=5), relative to the expression of dG counterparts (8-oxodG/G).
